# Antenatal thymus volumes in fetuses that delivered <32 weeks' gestation: An MRI pilot study

**DOI:** 10.1111/aogs.13983

**Published:** 2020-09-24

**Authors:** Lisa Story, Tong Zhang, Alena Uus, Jana Hutter, Alexia Egloff, Deena Gibbons, Alison Ho, Mudher Al-Adnani, Caroline L. Knight, Iakovos Theodoulou, Maria Deprez, Paul T. Seed, Rachel M. Tribe, Andrew H. Shennan, Mary Rutherford

**Affiliations:** 1 Department of Women and Children’s Health, School of Life Sciences, King’s College London, London, UK; 2 Fetal Medicine Unit, St Thomas’ Hospital, London, UK; 3 Artificial Intelligence Research Center, Peng Cheng Laboratory, Shenzhen, China; 4 Centre for the Developing Brain and Centre for Medical Engineering, King’s College London, London, UK; 5 Department of Immunobiology, King’s College London, London, UK; 6 Cellular Pathology Department, St Thomas’ Hospital, London, UK; 7 King’s College London Medical School, London, UK

**Keywords:** fetal thymus, infection, magnetic resonance imaging, preterm birth, thymus, volume

## Abstract

**Introduction:**

Infection and inflammation have been implicated in the etiology and subsequent morbidity associated with preterm birth. At present, there are no tests to assess for fetal compartment infection. The thymus, a gland integral in the fetal immune system, has been shown to involute in animal models of antenatal infection, but its response in human fetuses has not been studied. This study aims: (a) to generate magnetic resonance imaging (MRI) -derived fetal thymus volumes standardized for fetal weight; (b) to compare standardized thymus volumes from fetuses that delivered before 32 weeks of gestation with fetuses that subsequently deliver at term; (c) to assess thymus size as a predictor of preterm birth; and (d) to correlate the presence of chorioamnionitis and funisitis at delivery with thymic volumes in utero in fetuses that subsequently deliver preterm.

**Material and methods:**

Women at high-risk of preterm birth at 20-32 weeks of gestation were recruited. A control group was obtained from existing data sets acquired as part of three research studies. A fetal MRI was performed on a 1.5T or 3T MRI scanner: T2 weighted images were obtained of the entire uterine content and specifically the fetal thorax. A slice-to-volume registration method was used for reconstruction of three-dimensional images of the thorax. Thymus segmentations were performed manually. Body volumes were calculated by manual segmentation and thymus:body volume ratios were generated. Comparison of groups was performed using multiple regression analysis. Normal ranges were created for thymus volume and thymus:body volume ratios using the control data. Receiver operating curves (ROC) curves were generated for thymus:body volume ratio and gestation-adjusted thymus volume centiles as predictors of preterm birth. Placental histology was analyzed where available from pregnancies that delivered very preterm and the presence of chorioamnionitis/funisitis was noted.

**Results:**

Normative ranges were created for thymus volume, and thymus volume was standardized for fetal size from fetuses that subsequently delivered at term, but were imaged at 20-32 weeks of gestation. Image data sets from 16 women that delivered <32 weeks of gestation (ten with ruptured membranes and six with intact membranes) and 80 control women that delivered >37 weeks were included. Mean gestation at MRI of the study group was 28+4 weeks (SD 3.2) and for the control group was 25+5 weeks (SD 2.4). Both absolute fetal thymus volumes and thymus:body volume ratios were smaller in fetuses that delivered preterm (*P* < .001). Of the 16 fetuses that delivered preterm, 13 had placental histology, 11 had chorioamnionitis, and 9 had funisitis. The strongest predictors of prematurity were the thymus volume Z-score and thymus:body volume ratio Z-score (ROC areas 0.915 and 0.870, respectively).

**Conclusions:**

We have produced MRI-derived normal ranges for fetal thymus and thymus:body volume ratios between 20 and 32 weeks of gestation. Fetuses that deliver very preterm had reduced thymus volumes when standardized for fetal size. A reduced thymus volume was also a predictor of spontaneous preterm delivery. Thymus volume may be a suitable marker of the fetal inflammatory response, although further work is needed to assess this, increasing the sample size to correlate the extent of chorioamnionitis with thymus size.

## 1 Introduction

Preterm birth (PTB) defined as delivery at <37 weeks of gestation is a significant health burden projected to cost health services in England and Wales £2.946 billion (€3.279 billion) per year.^1^ The most significant adverse outcomes occur in very preterm deliveries, before 32 weeks of gestation, accounting for 1.4% of all deliveries in the UK.^2^

Although the etiology of PTB is complex and often multifactorial, infection/inflammation has been implicated, particularly at earlier gestations: at 28 weeks of gestation approximately 80% of cases of PTB have evidence of significant microbial colonization within the placental parenchyma.^3^ Neonatal morbidity, including sepsis, cystic periventricular leukomalacia, intraventricular hemorrhage, and later development of cerebral palsy, are significantly higher among preterm pregnancies that are also complicated by infection as assessed by the presence of chorioamnionitis at delivery.^4,5^ At present, there is no test routinely used in clinical practice to assess for fetal compartment infection and iatrogenic delivery, particularly for preterm premature rupture of the membranes (PPROM), is usually only instigated following signs of maternal infection, by which stage fetal infection may already be established.

The thymus plays an integral role in the development of the fetal immune system, being the main site of T-cell production and development, and has been suggested as a marker of the fetal inflammatory response,^6^ with involution occurring in the presence of acute stress, including malnutrition, trauma and sepsis.^7^ Although thymus size has previously been assessed using ultrasound^8^ and a reduction in length and perimeter has been associated with the presence of funisitis in pregnancies complicated by PPROM,^6^ adequate visualization can be hampered when ultrasound is used for assessment because of unfavorable fetal position, oligohydramnios, and high maternal body mass index.^6^ Ultrasound measurements in previous studies of pregnancies at high risk of preterm delivery have also only assessed two-dimensional markers such as the perimeter^6^ or cardiothorax:thymus ratios,^9^ which do not take into account variations in the true size and shape of the gland, and may not correlate with actual volume. Calculation of volumes of the gland using magnetic resonance imaging (MRI) obviates some of these difficulties and has successfully been used to evaluate the fetal thymus, both in uncomplicated pregnancies and in those with fetal growth restriction.^10^ More recent MRI advances have enabled reconstruction of the fetal thorax to further account for unpredictable fetal motion, so improving the accuracy of volumetric assessments.^11^

This study aims to investigate if alterations in thymus volume occur in fetuses that are delivered preterm and if there are associations between the size of the gland and the presence of chorioamnionitis at delivery. This will be achieved by: (a) generating MRI-derived normal ranges for thymus volume, and thymus volume standardized for fetal size, in uncomplicated pregnancies between 20 and 32 weeks of gestation; (b) comparing thymus size in fetuses that deliver very preterm, standardizing for fetal body volume; (c) assessing thymus size as a predictor of PTB; and (d) correlating the presence of chorioamnionitis and funisitis at delivery with thymic volumes in utero in fetuses that subsequently deliver preterm.

## 2 Material and Methods

This study was conducted at a large London teaching NHS Foundation Trust hospital. Women at high risk of preterm delivery were prospectively recruited with written informed consent between December 2015 and March 2020 (not all eligible women were approached during this time period, only during periods when the study team was recruiting).

Inclusion criteria were: gestational age 20-32 weeks and high-risk of PTB. This included either asymptomatic women with a history of previous PTB, late miscarriage >16 weeks, or cervical surgery with a >50% risk of PTB in the next 2 weeks (based on an algorithm derived from quantitative cervico-vaginal fibronectin and cervical length^12^) or women with PPROM, confirmed on clinical assessment at vaginal speculum examination. High-risk asymptomatic women were recruited from the Preterm Surveillance Clinic and women with PPROM were recruited from the antenatal ward or Maternity Assessment Unit. Exclusion criteria were: fetuses known to have structural or chromosomal abnormalities, multiple pregnancies, active labor, maternal inability to give informed consent, pregnancy complications including preeclampsia and fetal growth restriction, and contraindications to MRI such as claustrophobia or a recently sited metallic implant.

Following assessment of eligibility, women were invited to participate and written consent was obtained. A fetal MRI was performed using a 1.5T or 3T MRI System (Philips Achieva; Philips Medical Systems) with a 32-channel coil placed around the mother's abdomen. Imaging of the entire uterus to include whole fetal body and thorax was performed: T2 weighted single-shot turbo spin echo (ssTSE) images in three or more orthogonal planes re-orientated with respect to the fetus/uterus were acquired. The following scanning parameters were used: TR = 25 991 ms, TE = 80 ms, slice thickness of 2.5 mm, slice overlap of 1.25 × 1.25 × 1.25 mm on 1.5T, and 1.21 × 1.21 × 1.5 or 1.25 × 1.25 × 2.5 mm on 3T, with a flip angle = 90°. Three-dimensional (3D) MR images of the fetal thorax were obtained from 4-8 motion corrupted T2 MRI stacks using the deformable slice-to-volume reconstruction method.^13–15^ Scan length did not exceed 1 hour.

**Figure 1:**
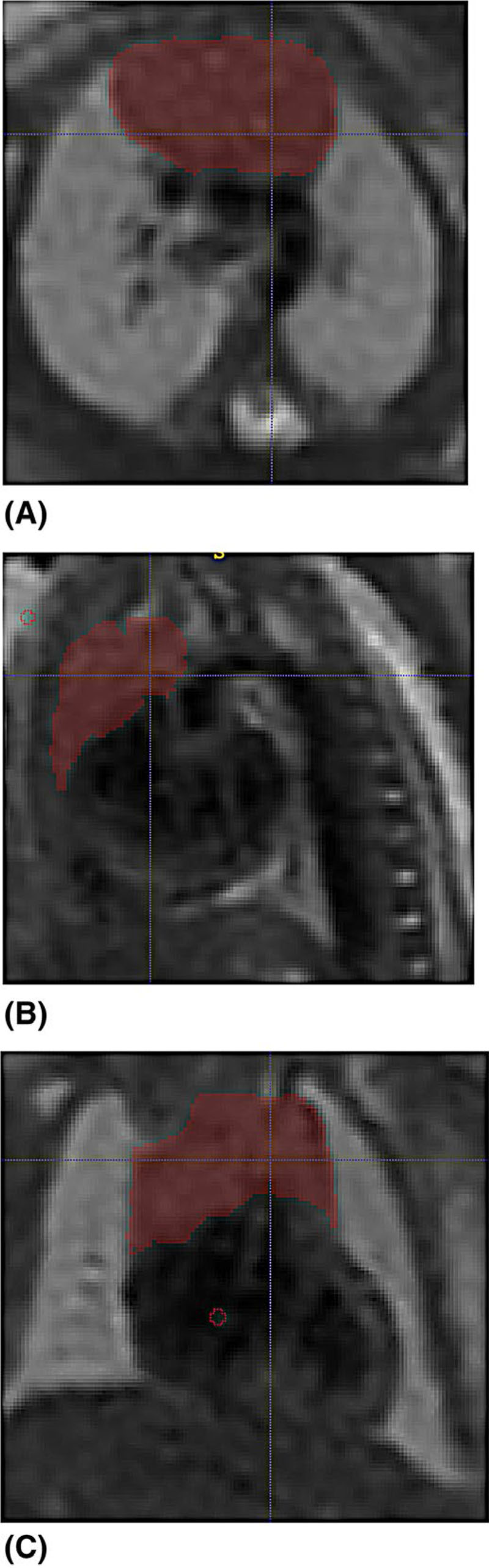
Thymus segmentation of a control fetus, at 30+0 weeks of gestation, that was subsequently delivered at term acquired on a 1.5T scanner in the (A) axial, (B) sagittal, and (C) coronal planes. The thymus is denoted in red [Color figure can be viewed at wileyonlinelibrary.com]

The fetal thymus, a bi-lobed structure located in the thorax anterior to the pericardium and posterior to the sternum, was segmented manually from the reconstructed images of the thorax using ITK-SNAP (version 3.6.0 http://itksnap.org)^16^ and 3D Slicer (version 4.10.2 https://www.slicer.org)^17^ (Figure 1). Intra- and inter-observer variability was confirmed by two operators (LS and AE). Semi-automatic segmentation of the fetal body was again performed using ITK-SNAP in a two-step process. Automatic segmentation of the body was based on image contrast while using user-defined thresholds. Fine editing of each segmentation was performed manually to remove incorrectly labeled areas. Good intra- and inter-observer variability had previously been confirmed for body volume calculation.^11^

A control group was identified from three other studies: the intelligent Fetal Imaging and Diagnosis project (www.iFINDproject.com), the Placental Imaging Project (www.placentaimagingproject.org) and ‘Antenatal assessment of fetal infection utilizing advanced MRI protocols’. All women had given informed written consent to allow their imaging data to be used. Cases were selected from uncomplicated pregnancies where the MRI was performed between 20^+0^ and 32^+0^ weeks of gestation and delivery occurred >37 weeks of gestation. All fetuses had undergone the same imaging protocol described above on a 1.5 Tesla or 3 Tesla scanner (Phillips) and had thymus and body volumes reconstructed using the same protocols.

Details of maternal demographics, delivery, and neonatal parameters were collected including: gestation at delivery, sex of infant, birthweight, birthweight centile, neonatal unit admission, number of days of invasive ventilation, continuous positive airway pressure, abnormalities on neurological imaging, and confirmed sepsis. In the preterm cohort, placentas were sent for a structured histological assessment^18^ as for routine clinical practice and more specifically to detect the presence of chorioamnionitis.^19^

### 2.1 Statistical analyses

Data were assessed for normality using distributional plots of standardized residuals. Demographic and neonatal outcome data were analyzed using a Student t test where data were continuous and chi-squared test where the data were categorical. For the control cases, the Wright and Royston xrimel method was used to estimate normal growth trajectory of thymus volume and thymus:body volume ratios between 20 and 32 weeks of gestation.^20^ Receiver operating curves (ROC) were generated for low thymus:body volume ratio and low gestation adjusted thymus volume centiles as predictors of PTB.

We estimated the difference between fetuses that delivered preterm and those that delivered at term in thymus and body volumes and thymus:body volume ratios adjusting for the effects of gestation and the strength of the magnet by multiple regression.

### 2.2 Ethical approval

This study was conducted under the ethics numbers 07/H0707/105 (dated 29 October 2007), 14/LO/1806 (dated 25 January 2015), 19/SS/0032 (dated 7 March 2019). All regulatory approvals were obtained before commencing the research.

## 2 Results

**Figure 2:**
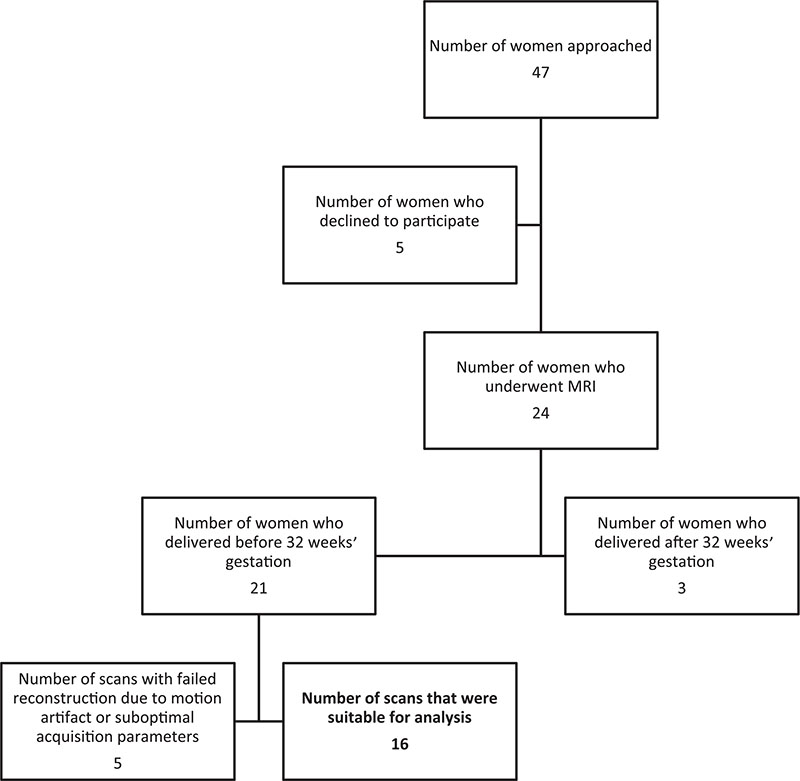
Flow chart illustrating study recruitment

Recruitment for this study can be seen in Figure 2. Of the 16 pregnancies analyzed, 13 of the women had received steroids for fetal lung maturity before the time of imaging. Ten of the women had ruptured membranes at the time of MRI and six had intact membranes. Ten examinations were performed on a 1.5T scanner and six on a 3T scanner. Of the 16 fetuses that delivered preterm, 13 had placental histology available. Of these, 11 had evidence of chorioamnionitis in the placenta and nine had funisitis.

In addition, 80 control cases were identified. All of these pregnancies were delivered after 37 weeks of gestation and were scanned on a 1.5T (50 cases) or 3T (30 cases) scanner using similar protocols: 3D reconstruction of the fetal body was performed for each of the data sets.

**Figure 3:**
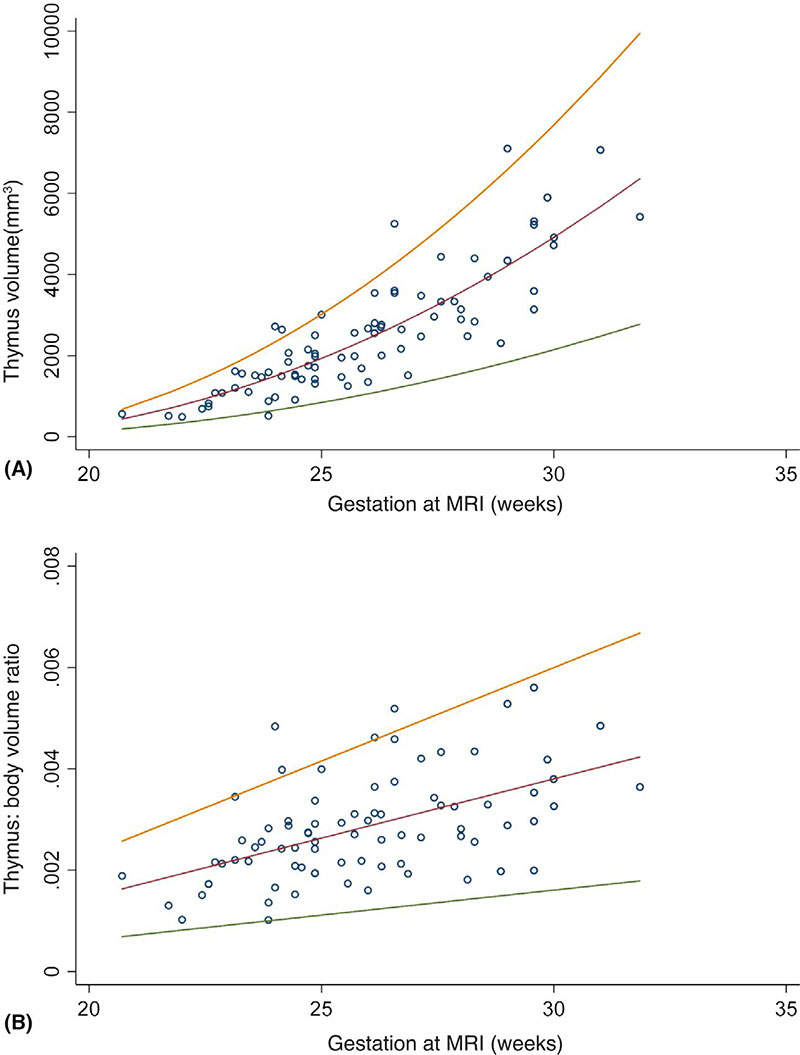
Magnetic resonance imaging (MRI) -derived normal ranges of fetal thymus volumes between 20 and 32 weeks of gestation from healthy pregnancies that subsequently delivered at term. Normal ranges and 3rd and 97th centiles. The expected mean thymus volume (mm^3^), Z score and gestation adjusted centile at a given gestation age (GA) were estimated: *X =* GA/10 -2.612052579; *m* = -8716.91 × *X* + 2670.851 × *X*^2^ + 2480.724; *s* = 0.2998746 × *m*; and Z-score = (thymus volume - m)/s. The expected mean thymus:body volume ratio, Z score and gestation adjusted centile at a given GA were estimated: *X* = GA -26.12052579; *m* = 0.002338 × *X* + 0.0028938; *s* = 0.3070776 × *m*; and Z-score = (thymus:body volume ratio - m/s) [Color figure can be viewed at wileyonlinelibrary.com]

Clinical characteristics of the group that delivered preterm and the control group are denoted in Table 1. Distribution plots can be seen for thymus volumes and thymus:body volume ratios from 20 to 32 weeks of gestation derived from the control group of fetuses in Figure 3.

**Figure 4:**
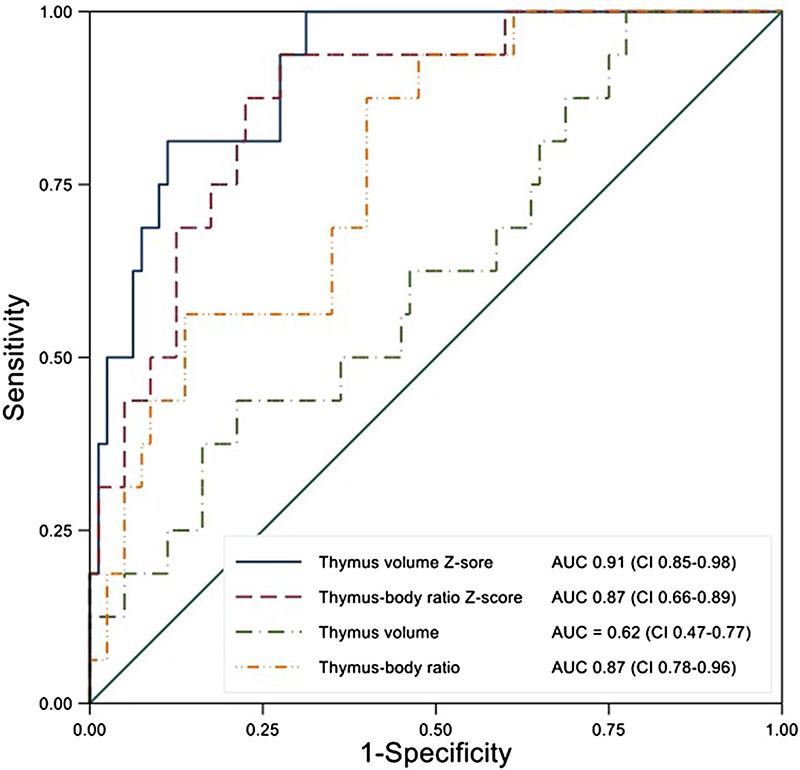
Receiver operator curve prediction of prematurity from antenatal magnetic resonance imaging scans: thymus volume, thymus:body volume ratio, thymus volume Z-score, and thymus:body volume ratio Z-score including 95% confidence intervals [Color figure can be viewed at wileyonlinelibrary. com]

**Figure 5:**
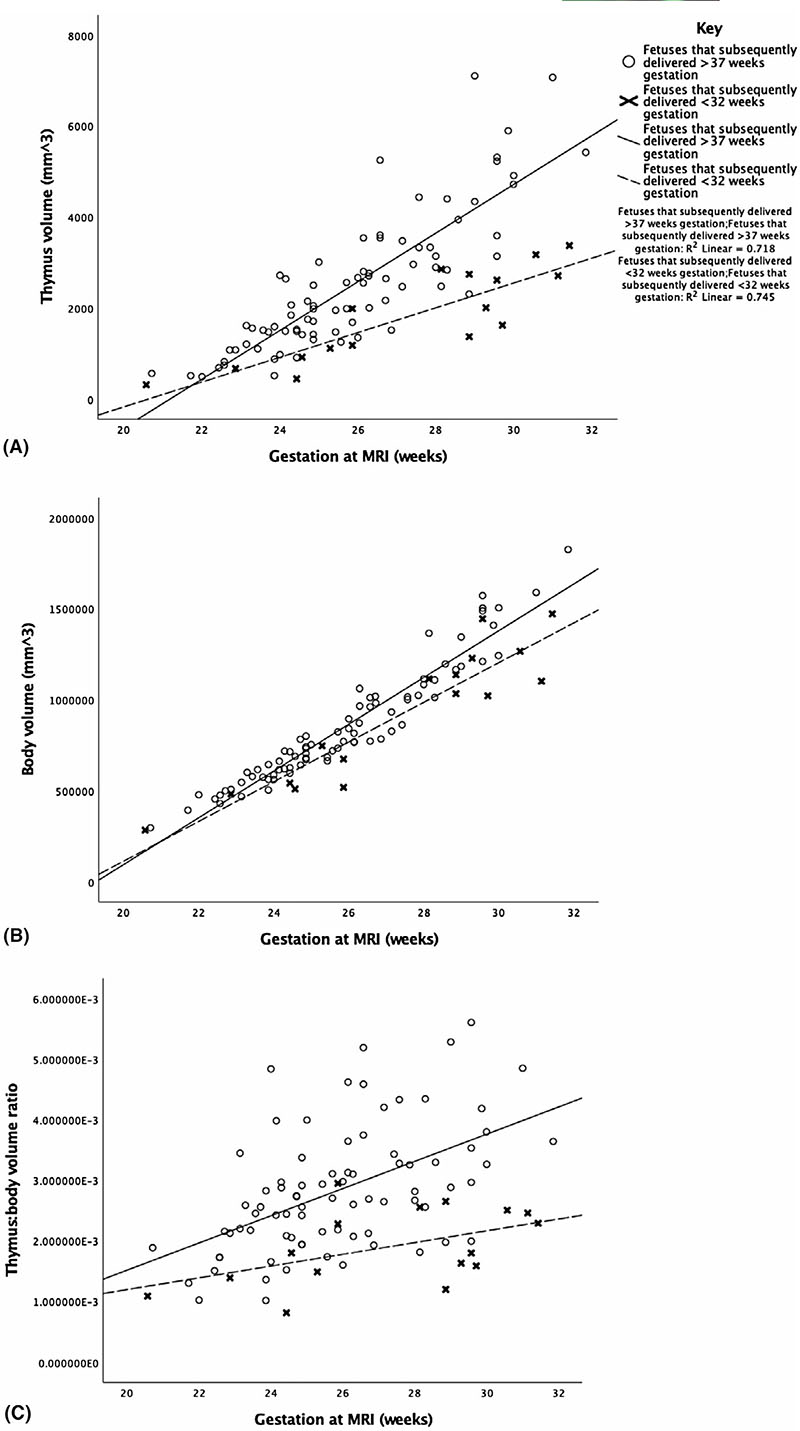
Graphs illustrating the antenatal thymus (A) and body (B) volumes and the thymus:body volume ratios (C) between 20 and 32 weeks of gestation in fetuses that delivered before 32 weeks of gestation and those that delivered at term

The gestation-adjusted thymus volume centiles and the thymus:body volume ratio were tested as predictors of PTB (Figure 4). Fetuses that delivered preterm had significantly lower thymus volumes and body volumes at the time of scan than those that delivered at term, accounting for the effects of gestation (Table 2; Figure 5). When standardized for fetal size, fetuses that delivered preterm had significantly lower thymus:body volume ratios. No effect was demonstrated regarding magnet strength on these findings. As previously reported, fetal body volumes were also significantly smaller in fetuses that delivered preterm (*P* < .001).^11^ Although numbers are small the finding that thymus volume and thymus:body volumes were smaller persisted in the preterm cohort in both fetuses with ruptured membranes and those with membranes intact.

**Figure 6:**
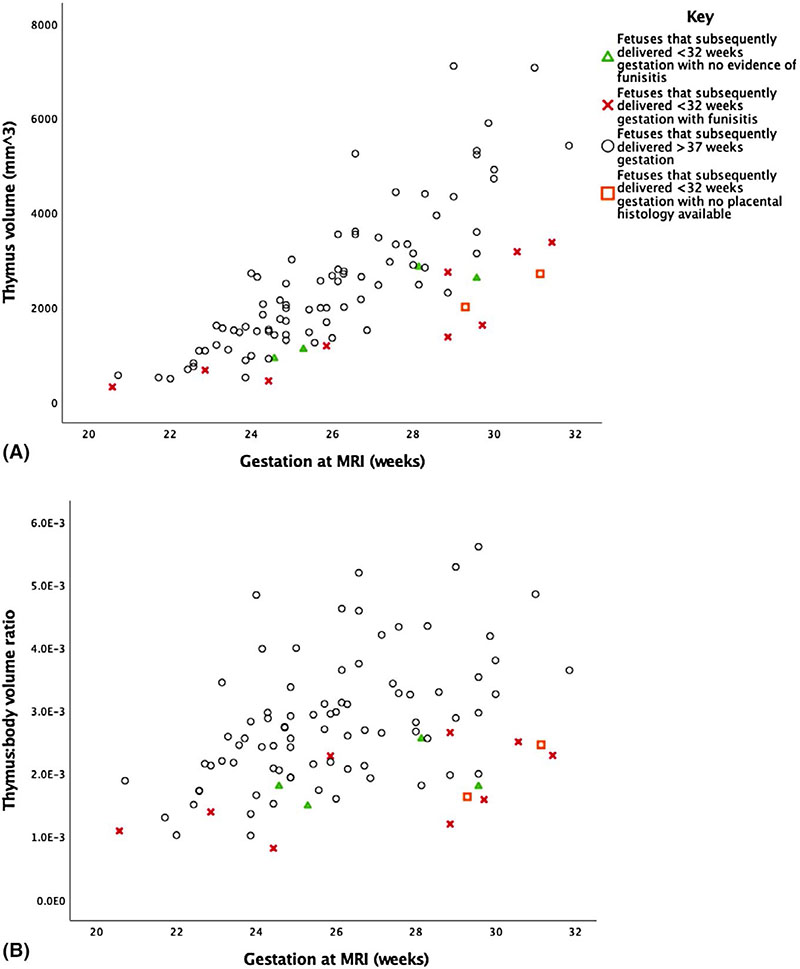
Graphs illustrating the antenatal thymus (A) and the thymus:body volume ratios (B) between 20 and 32 weeks of gestation in fetuses that delivered before 32 weeks of gestation, with and without funisitis at delivery and those that delivered at term [Color figure can be viewed at wileyonlinelibrary.com]

Although the numbers are small, the relation between thymus volume and the presence of funisitis can be seen in Figure 6.

There was one intrapartum death at 22^+0^ weeks of gestation with a reduced thymus:body volume ratio of 0.011. The mean number of days from MRI to delivery in the preterm group was 10.5 (SD 14.2). Two fetuses from the control group that delivered >37 weeks were admitted to the neonatal unit, one for a few hours only with suspected respiratory distress syndrome and the second for 2 days with hypoglycemia.

## 4 Discussion

We have reported normal ranges for MRI-derived fetal thymus volume between 20 and 32 weeks of gestation, finding that absolute thymus volumes and thymus:body volume ratios increase with gestation in uncomplicated pregnancies. We have also demonstrated a smaller thymus volume in fetuses that subsequently deliver very preterm. This finding persists when thymus volume is standardized for the overall body volume of the fetus. Where histology was available, 85% of the preterm cases showed evidence of chorioamnionitis and 69% had evidence of funisitis.

The findings, of a small thymus in fetuses with PPROM, are in accordance with previous US studies. Associations between a reduction in length of the fetal thymic perimeter, <5th centile, measured on an axial 2D ultrasound image, and chorioamnionitis,^21^ funisitis,^6^ and elevated cord blood interleukin-6 levels at delivery^6^ have previously been reported in women with PPROM. Assessment of the transverse diameter of the thymus has also been investigated as a predictor of neonatal sepsis, a measurement <5th centile having a sensitivity of 100%, specificity of 73%, positive predictive value of 55%, and negative predictive value of 100% in detecting subsequent early neonatal sepsis.^22^

A reduction in fetal thymus perimeter has also been found in women at high risk of PTB but with intact membranes, which correlated with the presence of both intrauterine infection and chorioamnionitis at delivery.^23^ The thymus diameter was assessed using 2D ultrasound, and amniocentesis was performed to assess for microbial invasion of the amniotic cavity. Placentas were then assessed for histological evidence of chorioamnionitis at delivery; 32.3% of women had intra-amniotic infection and 51.6% had evidence of placental chorioamnionitis. Isolated histological chorioamnionitis and funisitis were found in 22.6% and 25.8% of fetuses, respectively. In all cases of intrauterine infection and 23.8% of cases without intrauterine infection, the fetal thymus perimeter was <5th centile for gestational age. The fetal thymus was <5th centile for gestational age in 100%, 71.4% and 12.5% of women with histological signs of funisitis and isolated chorioamnionitis, and without histological signs of infection, respectively.^23^ This again is in accordance with this study's findings of a smaller thymus size in fetuses that subsequently deliver preterm. The numbers in this study are too small to find exact associations between the thymus volume and the presence of chorioamnionitis; however, there was a trend towards a smaller thymus in fetuses delivering very preterm with evidence of funisitis.

In contrast to these findings, Brandt et al prospectively assessed the fetal thymus in 520 pregnancies using 2D ultrasound, measuring the transverse and anterio-posterior thymus diameters and the thymic:thoracic ratio. Of this population, 12.3% underwent PTB; however, there was no correlation between thymus size and premature delivery.^9^ It should be noted that the median gestational age at imaging was 20.5 weeks and very few of the PTBs occurred very early in gestation, hence the infective/inflammatory processes may not have commenced at the time of this initial scan.

All of the previous studies, however, have only evaluated the thymus using two-dimensional parameters. Its shape is variable and volumetric assessment provides a more complete evaluation of the size of the gland because of its lobular nature. A previous study by Li et al compared 2D and 3D ultrasound measurements of the thymus, finding that the correlation between thymus volume and gestational age was significantly stronger using 3D volume measurement than that of any of the 2D parameters evaluated.^24^

Although thymic volume has previously been assessed using 3D ultrasound in uncomplicated pregnancies^25,26^ and in those with growth restriction,^10,27^ to our knowledge our study is the first to assess thymus volume, using either modality, MRI or ultrasound, in pregnancies at high risk of PTB. MRI may be a superior imaging modality in this cohort of fetuses, particularly those with ruptured membranes as oligohydramnios and unfavorable fetal lie can give inadequate visualization of the gland.^6^

Although further work is required to assess the relation in more detail, thymus volume may be reduced in fetuses at high risk of PTB because of alterations in the structure of the gland occurring in the presence of fetal compartment infection. Toti et al undertook histological assessment of the thymus in a number of late miscarriages and PTBs resulting in fetal demise/neonatal death due to sepsis with evidence of histological chorioamnionitis (n = 40).^7^ Where there was evidence of chorioamnionitis, the thymus revealed more advanced signs of cortical shrinkage and lobules were more separated, confirmed by fractal dimension analysis. Although there was variation within the group this was thought to be attributable to the differences in the length of exposure to in utero infection. Term and preterm neonates who died of sepsis all had thymuses that were almost completely devoid of thymocytes and with irregular narrowing of the cortex.

In contrast, in a sheep model, following lipopolysaccharide-induced chorioamnionitis, no histological changes in the thymus or changes in markers of apoptosis and proliferation in the thymoctes were identified. However, as was the case in our study, thymus:body-weight ratios were reduced by 40% 5 days after lipopolysaccharide administration. Blood lymphocytes were also found to be 40% lower than the control group after 1 day and elevated at 5 days.^28^ However, these findings, and the lack of histological changes in the gland, may be attributable to the fact that the sheep model gave a single dose of lipopolysaccharide whereas the antenatal insult may be more extensive and prolonged in clinical practice.

At present, numbers within this current study are small, the timing between the MRI scan and delivery varies, and placental histology was not available in all cases. The slight difference in the gestation at which the MRI was performed between cases and controls is a limitation of the study; however, it has been adjusted for in the analysis. Furthermore, the presence of confounding factors such as the timing of steroid administration, which has been shown to alter the structure of the gland in an ovine model of chorioamnionitis, needs to be considered in a larger sample.^29^ Further work is also required to correlate the imaging findings with markers of infection in both placental histology and umbilical cord blood and both short-term and long-term postnatal outcomes. In this study, the thymus was not the primary focus of all of the investigations and in 24% of cases a thymus volume could not be reconstructed. Acquisition techniques have been optimized and we would anticipate this percentage being significantly lower in the future. In addition, MRI studies in the future could include additional sequences to detect microstructural and perfusion alterations within the thymus that may also be associated with a reduction in volume and the presence of funisitis.

## 5 Conclusion

We have reported normal ranges, between 20 and 32 weeks of gestation, for thymus volume and thymus volume standardized for fetal size derived from healthy fetuses that subsequently delivered at term. Thymus volumes are smaller in fetuses that subsequently deliver very preterm, a finding which persisted after standardization for fetal size. Although numbers were too small to compare fetuses with and without chorioamnionitis/funisitis at delivery it may be reflective of thymic involution as a consequence of infective/inflammatory processes in the in utero environment, which are also associated with the etiology of preterm delivery. Thymus volume and thymus:body volume ratio also appeared to be good predictors for preterm delivery. Further evaluation is needed to assess the utility of thymus volume as a marker for the fetal inflammatory response both using MRI and using ultrasound. MRI potentially has the advantage over ultrasound as it can provide visualization of the whole gland, particularly in pregnancies where ultrasound images may be suboptimal such as those involving oligohydramnios, raised body mass index, and unfavorable fetal position, and can provide more insight into tissue microstructure and perfusion. Evaluation of the thymus shows potential to help to inform the timing of delivery where there is clinical uncertainty regarding the presence of fetal compartment infection. It may also serve as a marker of fetal compartment infection in pregnancies where there has been instrumentation of the uterine cavity, such as in cases of fetal surgery.

## Figures and Tables

**Table 1:** Clinical characteristics of the cohort (*t* test used for analysis where data were continuous and chi-squared test where data were categorical)

Characteristic	Preterm cohort (n = 16)	Term cohort (n = 80)	*P*	95% CI
Maternal age (y)				
Mean (SD)	37 (5.7)	33 (4.1)	.09	-5.0 to -0.2
BMI (kg/m^2^)				
Mean (SD)	23 (2.6)	22.9 (3.0)	.51	-2.5 to 1.7
Ethnicity, n (%)				
White	8 (50)	59 (77)		
Black	3 (19)	11 (14)		
South Asian	3 (19)	1 (1)		
East Asian	1 (6)	6 (2)		
Other	1 (6)	5 (8)		
Ethnicity grouping, n (%)				
White	8 (50)	59 (77)	.157	
Non white	9 (50)	23 (33)		
Parity, n (%)				
Primiparous	8 (50)	54 (68)	.13	
Multiparous	8 (50)	25 (32)		
GA at MRI, wk				
Mean (SD)	28.9 (3.2)	25.7 (2.4)	.04	-2.8 to -0.1
GA at birth, wk				
Mean (SD)	29.7 (2.4)	40.1 (1.2)		
Birthweight, g				
Mean (SD)	1285 (319)	3420 (471)	<.001	1836-2347
Range	770-1875	2540-4560		
Birthweight centile of live	births			
0-3	1 (7)	3 (4)	.94	
3-10	0	5 (6)		
10-25	3 (20)	17 (21)		
25-50	4 (27)	15 (19)		
50-75	5 (33)	22 (28)		
75-90	1 (6)	12 (15)		
90-97	1 (6)	6 (8)		
97-100	0	1 (2)		
Sex of infant, n (%)				
Female	6 (56)	38 (48)	.4	
Male	9 (36)	42 (53)		
Undetermined	1 (6)	0		
Outcome, n (%)				
Live to discharge	15 (94)	80 (100)	.2	
Neonatal/ intrapartum death	1 (6)			

Abbreviations: BMI, body mass index; GA, gestational age; MRI, magnetic resonance imaging.

**Table 2:** Thymus and body volumes in fetuses that delivered preterm and those that delivered preterm controlling for the effect of gestational age at magnetic resonance imaging scan by multiple regression

Variable	Term cohort, mean (SD) (n = 80)	Preterm cohort, mean (SD) (n = 16)	Difference in preterm cohort (95% CI)	*P*
Thymus volume (mm^3^)	2513 (1500)	1817 (1003)	-1366 (-1823 to -909)	<.001
Body volume (mm^3^)	850 381 (319 547)	911 130 (374 058)	-120 252 (-175 693 to -64 811)	<.001
Thymus:Body volume ratio	0.0028 (0.001)	0.0019 (0.0006)	-0.001 (-0.002 to -0.001)	<.001

## Key message

Thymus volumes are smaller in fetuses that deliver very preterm when standardized for fetal size. This may represent a marker of the fetal inflammatory response.

## Abbreviations

MRI: magnetic resonance imaging
PPROM: preterm premature rupture of membranes
PTB: preterm birth
ROC: receiver operating curves
